# Antioxidant and Sensory Assessment of Innovative Coffee Blends of Reduced Caffeine Content

**DOI:** 10.3390/molecules27020448

**Published:** 2022-01-10

**Authors:** Danijela Šeremet, Patricia Fabečić, Aleksandra Vojvodić Cebin, Ana Mandura Jarić, Robert Pudić, Draženka Komes

**Affiliations:** 1 Faculty of Food Technology and Biotechnology, University in Zagreb, Pierottijeva Street 6, 10000 Zagreb, Croatia; dseremet@pbf.hr (D.Š.); pfabecic@pbf.hr (P.F.); avojvodic@pbf.hr (A.V.C.); amandura@pbf.hr (A.M.J.); 2 Quahwa Coffee Roastery & Coffee Shop, Nikola Tesla Street 9/1, 10000 Zagreb, Croatia; robert.pudic@quahwa.hr

**Keywords:** antioxidants, caffeine, coffee, sensory evaluation

## Abstract

Considering the current trend in the global coffee market, which involves an increased demand for decaffeinated coffee, the aim of the present study was to formulate coffee blends with reduced caffeine content, but with pronounced antioxidant and attractive sensory properties. For this purpose, green and roasted Arabica and Robusta coffee beans of different origins were subjected to the screening analysis of their chemical and bioactive composition using standard AOAC, spectrophotometric and chromatographic methods. From roasted coffee beans, espresso, Turkish and filter coffees were prepared, and their sensory evaluation was performed using a 10-point hedonic scale. The results showed that Arabica coffee beans were richer in sucrose and oil, while Robusta beans were characterized by higher content of all determined bioactive parameters. Among all studied samples, the highest content of 3-*O*-caffeoylquinic acid (14.09 mg g^−1^ dmb), 4-*O*-caffeoylquinic acid (8.23 mg g^−1^ dmb) and 5-*O*-caffeoylquinic acid (4.65 mg g^−1^ dmb), as well as caffeine (22.38 mg g^−1^ dmb), was detected in roasted Robusta beans from the Minas Gerais region of Brazil, which were therefore used to formulate coffee blends with reduced caffeine content. Robusta brews were found to be more astringent and recognized as more sensorily attractive, while Arabica decaffeinated brews were evaluated as more bitter. The obtained results point out that coffee brews may represent a significant source of phenolic compounds, mainly caffeoylquinic acids, with potent antioxidant properties, even if they have reduced caffeine content.

## 1. Introduction

Coffee is one of the most consumed beverages worldwide with the largest segment in the market of hot drinks, which is expected to generate global revenue of USD 585 billion by 2025 [1]. The most popular coffee varieties are Robusta (*Coffea canephora*) and Arabica (*Coffea arabica*). Arabica coffee is generally more accepted by consumers due to its sweeter taste, fruity aroma and higher acidity, while Robusta coffee is characterized by bitterness and less acidity and sweetness, and is therefore most commonly used in blends with Arabica-based coffee or made into instant coffee [2]. Global production of Arabica coffee was 102.1 million 60 kg bags in 2020/21, with a decline expected in the following marketing year [3]. Robusta coffee, on the other hand, whose production amounted 73.72 million 60 kg bags during the same period, is predicted to grow to over 77 million 60 kg bags [4]. Brazil, Vietnam, Colombia and Indonesia stand out as the largest coffee producers [5]. 

Most consumers drink coffee for its unique sensory properties, i.e., taste and smell, which trigger positive feelings, as well as for physical and cognitive stimulation, or simply out of habit as the part of daily ritual [6]. Much epidemiological evidence to date links habitual coffee consumption to several health benefits, such as reduced risk of Parkinson’s and Alzheimer´s disease, type 2 diabetes, endometrial, prostate, colorectal and liver cancers, a beneficial effect on liver function and a possible role in weight loss [7]. The main pharmacologically active compound in coffee is caffeine (1, 3, 7-trimethylxanthine), a natural alkaloid found in more than 60 plants such as tea leaves, kola nuts and cocoa pods, but coffee is its primary source in all consumer age groups [7]. Caffeine binds to the receptors of the neuromodulator adenosine, which promotes sleep and affects memory and learning. Its absorption in the small intestine is rapid and complete in less than 1 h with rapid diffusion to other tissues. The half-life of caffeine in adults is 3–7 h and it is metabolized primarily in the liver [8]. According to EFSA [9], caffeine intake from all caffeinated sources up to 400 mg per day consumed throughout the day does not cause safety concerns for healthy adults in the general population. Although it is hard to reach an upper limit of daily caffeine consumption [10], it has been reported that even moderate ingestion of caffeine for some consumers could be related to negative side effects, such as increased blood pressure, insomnia or anxiety disorders. It is important to emphasize that the mentioned downsides and decreased tolerability are dependent on dose, frequency of intake and probably associated with individual genetic variability [11,12]. Due to consumers’ awareness of adverse physiological effects of caffeine and caution not to exceed the recommended daily intake without abstaining from the coffee consumption, the global decaffeinated coffee market size is growing, with a value of USD 1.65 billion in 2019, and is predicted to grow further [13]. Many consumers switch from caffeinated to decaffeinated coffee due to illness or symptoms [14], while the importance of health-promoting lifestyles for the currently most important segment of the consumer population, i.e., millennials, indicates movement toward a significant increase in functional and decaffeinated beverages on the market share [15]. Decaffeinated coffee used to be made from lower quality beans and the used processes significantly altered the flavor profile, resulting in quite poor sensory characteristics of these brews. Today, however, modern decaffeination processes have reached a professional and satisfactory level, so that the main bioactive and aromatic compounds in decaffeinated coffee beans are preserved by properly matching the roasting process to the decaffeinated beans, while vacuum drying and additional polishing processes can help improve a dull surface [16,17]. Decaffeination of coffee can be successfully accomplished by various methods, such as organic solvent-based decaffeination, the carbon method and supercritical CO_2_ decaffeination, or, to avoid additional processes, equipment and materials associated with the mentioned processes and low-caffeine coffee mutants or hybrids can be used [18]. Regarding caffeine, coffee represents a prominent source of bioactive compounds, primarily polyphenols, with dominant caffeoylquinic acids. In numerous studies conducted to date, phenolic compounds as antioxidative agents are linked to strong health-promoting benefits [19]. The qualitative and quantitative content of these physiologically active compounds is influenced by numerous factors, including coffee species and its geographical origin, storage and roasting conditions, the type of coffee brewing preparation, etc. [20,21,22,23], whereby various possibilities could be created to produce coffee or coffee blends with specific and prominent sensory and antioxidative attributes. 

Considering the increased global demand for decaffeinated coffee brews, the aim of the present study was to formulate coffee blends with pronounced antioxidant and attractive sensory properties, but with reduced caffeine content. For this purpose, Arabica and Robusta beans of different origins were analyzed for their chemical and bioactive composition and were subjected to sensory evaluation to formulate coffee blends with desirable properties, showing both sufficient antioxidant intake and potent sensory attributes.

## 2. Materials and Methods

### 2.1. Materials and Chemicals

#### 2.1.1. Materials

Species and region of origin of investigated coffee beans are presented in Table 1. 

Roasting was performed according to the parameters listed in Table 2 in the Giesen W30A coffee roaster (Giesen, Ulfit, The Netherlands). The parameters were selected in a “trial and error” approach for each coffee until a satisfactory aroma and flavor profile of the coffee was achieved. The roasting degree was medium for all coffee samples. 

#### 2.1.2. Chemicals

Petroleum ether was supplied from Kemika (Zagreb, Croatia). Folin–Ciocalteu reagent and sodium carbonate were supplied from Lach-ner (Neratovice, Czech Republic). Lead(II) acetate trihydrate, (*S*)-6-Methoxy-2,5,7,8-tetramethylchromane-2-carboxylic acid (Trolox), 2,2-Diphenyl-1-picrylhydrazyl (DPPH), 2,20-Azino-*bis*(3-ethylbenzothiazoline-6-sulfonic acid) diammonium salt (ABTS), gallic acid (>97%), chlorogenic acid/3-*O*-caffeoylquinic acid (3-CQA; 95%), cryptochlorogenic acid/4-*O*-caffeoylquinic acid (4-CQA; >98%) and neochlorogenic acid/5-*O*-caffeoylquinic acid (5-CQA; >98%) were purchased from Sigma-Aldrich (St. Louis, MO, USA). Standards of sucrose and caffeine were purchased from Fluka (Taufkirchen, Germany). Ethanol and sodium oxalate were supplied from Gram-mol d.o.o. (Zagreb, Croatia), and acetonitrile, methanol and formic acid from Fisher Scientific (Hampton, NH, SAD). Caramel was supplied by Frutarom (Haifa, Israel). All chemicals used for experimental procedures were of analytical or HPLC grade.

### 2.2. Methods

#### 2.2.1. Preparation of Coffee Samples

Green and roasted coffee beans were ground in an ultracentrifugal mill (ZM 200, Retsch, Germany) and as such were used for all further analyses. 

#### 2.2.2. Determination of Dry Matter, Oil and Sucrose Content

The dry matter was determined according to the AOAC 930.15 method [24] by drying the samples until constant mass at 105 °C. The crude oil content was determined according to the AOAC 920.39 method [25] using a Soxhlet extractor and petroleum ether as solvent. For the determination of sucrose, water extracts of samples (1 g of sample in 50 mL of water) were prepared in a water bath (Inko VKZ ERN, Inkolab d.o.o., Zagreb, Croatia) for 45 min at 90 °C. Upon the completion of extraction, centrifugation was performed (9500 rpm, 20 min). Supernatant was further transferred to a volumetric flask of 50 mL to which 1 mL of saturated solution of neutral lead acetate was added. The content of the flask was well mixed, filled to the mark with water and filtrated (Whatman^®^ filter paper 4). Sodium oxalate was added to precipitate the excess of lead used in the purification process. Extracts were once again filtered and used for HPLC analysis. Determination of sucrose was performed on a Hi-Plex Ca column (300 × 7.7 mm) and the Agilent 1200 Series chromatographic system (Agilent Technologies, Santa Clara, CA, USA) coupled with a refractive detector (Agilent Technologies, Santa Clara, CA, USA). The mobile phase was water. With respect to refractometric detection, isocratic elution of the analyte at a flow rate of 0.6 mL min^−1^ for 15 min was established. The temperature of the column was 80 °C and that of the detector 40 °C. The volume of injected samples was 10 μL. Sucrose identification was performed by comparing the retention time with a commercially available standard, while quantification was enabled by establishing a sucrose calibration curve (20–1000 µg mL^−1^). The analysis was performed in duplicate. All samples were filtered through a 0.45 µm membrane filter (Nylon Membranes, Supelco, Bellefonte, PA, USA) prior to the analysis. 

#### 2.2.3. Determination of Melanoidin Content

The same water extracts of roasted coffee beans prepared for the determination of sucrose (without the purification process) were subjected to the spectrophotometric analysis (Genesys 10S UV–Vis spectrophotometer, Thermo Fisher Scientific, Waltham, MA, USA) of melanoidins by monitoring the absorbance at 420 nm. The standard calibration curve was constructed with solutions of caramel (250–1000 µg mL^−1^) as the melanoidin standard [26]. The absorbance of 0.1% (*w*/*v*) caramel solution in water in a 1 cm cell at 610 nm was 0.145 [27]. The results were expressed as the percentage (%) of caramel melanoidins in the sample’s dry matter (% CM dmb). All measurements were performed in triplicate.

#### 2.2.4. Extraction of Phenolic Compounds and Caffeine

The ground coffee beans (1 g) were used to extract phenolic compounds and caffeine using 70% (*v*/*v*; 20 mL) ethanolic solution as solvent. Continuous double extraction was performed at 85 °C for 25 min on a magnetic stirrer (WiseStir SMHS-6, Witeg, Wertheim, Germany) with an aqueous reflux condenser. Extraction was followed by centrifugation (9500 rpm, 20 min) and filtration (Whatman^®^ filter paper 4). 

#### 2.2.5. Determination of Total Phenolic Content (TPC) and Antioxidant Capacity

Total phenolic content (TPC) in prepared ethanolic extracts was determined spectrophotometrically (Genesys 10S UV–Vis spectrophotometer, Thermo Fisher Scientific, Waltham, MA, USA) following the method of Singleton and Rossi [28]. The standard calibration curve was constructed with solutions of gallic acid (25–200 µg mL^−1^). The results were expressed as mg gallic acid equivalents/g of the sample’s dry matter (mg GAE g^−1^ dmb). The antioxidant capacity was determined using the DPPH and ABTS radical cation decolorization assays described by Brand-Williams et al. [29] and Re et al. [30], respectively. Solutions of Trolox (25–200 µmol mL^−1^) were used for the construction of the standard calibration curve, and the results were expressed as µmol Trolox equivalent/g of the sample’s dry matter (µmol TroloxE g^−1^ dmb). All measurements were performed in triplicate.

#### 2.2.6. Determination of Individual Phenolic Compounds and Caffeine

The HPLC analysis was performed on an Agilent Series 1200 chromatographic system (Agilent Technologies, Santa Clara, CA, USA) coupled with a photodiode array detector (PAD) (Agilent Technologies, Santa Clara, CA, USA) and using a Zorbax Extend C18 (4.6 × 250 mm, 5 μm i.d.) chromatographic column (Agilent Technologies, USA). The elution was performed in a gradient with a mobile phase consisting of (A) 1% (*v*/*v*) formic acid solution in water and (B) 1% (*v*/*v*) formic acid solution in acetonitrile. The regimen was as follows: 0 min—7% B; 5 min—7% B; 45 min—40% B; 47 min—70%; 52 min—70% B. The flow was 1 mL min^−1^, the injection volume 5 μL and the column temperature 25 °C. The chromatograms were recorded at 278 and 320 nm. The analysis for all samples was performed in duplicate. All samples were filtered through a 0.45 μm membrane filter (Nylon Membranes, Supelco, Bellefonte, PA, USA) prior to the analysis. Identification of individual phenolic compounds and caffeine was performed by comparing the retention times and the characteristic absorption spectrum (190–400 nm) with commercially available standards, while quantification was enabled by establishing calibration curves (20–100 µg mL^−1^).

#### 2.2.7. Preparation of Coffee Brews

Espresso was prepared in the Rocket Espresso Milano R60V (Rocket Espresso Milano, Milano Italy) with a 1 g:20 mL sample/solvent (water) ratio. Turkish coffee was prepared by bringing a mixture of coffee and water (1 g:10 mL) to a boil. Boiling was stopped when a foam formed on the surface of the brew. Filter brew was prepared by pouring hot water (92 °C) over coffee (1 g:15 mL) and then filtering [31].

#### 2.2.8. Sensory Analysis

Sensory analyses of espresso, Turkish and filter coffee brews was performed using a 10-point hedonic scale ranging from 1 (detectable) to 10 (very intense) [32]. The sensory panel consisted of 20 staff members (15 women and 5 men) from the Faculty of Food Technology and Biotechnology, University of Zagreb (Zagreb, Croatia) who had previous experience with sensory analysis of coffee brews. The aroma and flavor sensory attributes evaluated and graded by the panelists were roast, nutty, chocolate, bitter, astringent and acid. Panelists were asked to note whether they perceived any additional aroma or flavor (descriptive analysis). All samples were evaluated in partitioned booths of the sensory laboratory under white light illumination at room temperature. At least 25 mL of the sample was served in transparent glasses per panelist. Water was provided for rinsing between samples. 

##### Formulation of Coffee Blends with Reduced Caffeine Content

Considering the evaluated antioxidant capacity, the qualitative and quantitative results of the analyzed bioactive compounds of the roasted coffee beans and the results of the sensory analysis of the coffee brews, the formulation of the coffee blends was conducted. Three coffee blends were formulated consisting of 70% RC_6 and 30% RC_5, 70% RC_4 and 30% RC_6 and 50% RC_6 and 50% RC_1. The main aim was to formulate coffee blends with reduced caffeine content, maximally enriched with phenolic compounds with high antioxidant capacity, while possessing desirable sensory properties.

#### 2.2.9. Statistical Analysis

Single-factor ANOVA was performed in Microsoft Excel (Microsoft, Redmond, Washington, SAD) with a significance level of α = 0.05%. One-way ANOVA and post hoc analysis (Tukey’s HSD test) were performed using the SPSS Statistics 17.0 program (IBM, Armonk, NY, USA) with a significance level of α = 0.05%.

## 3. Results and Discussion

### 3.1. Chemical Composition of Green and Roasted Coffee Beans

Chemical composition of green and roasted coffee beans was evaluated in terms of dry matter (DM), oil and sucrose content. The obtained results are presented in Table 3.

DM of green coffee beans ranged from 89.54% (sample GC_4) to 91.68% (sample GC_3). After harvesting, coffee beans are processed as soon as possible by dry or wet processing to a moisture content of about 10–12% [33], which is in accordance with the presented results. Further processing includes the roasting process which reduces the water content to as low as 2.5% [33], although lower values were obtained in the present study. The values of DM for roasted coffee beans were in a narrow range between 98.65% (sample RC_5) and 99.52% (sample RC_6). The largest increase in DM (9.50%) after roasting was observed in sample GC_4 and could be explained by the fact that it initially had the lowest DM content as green beans, which further decreased during the roasting process due to the highest initial roasting temperature (430 F, Table 2).

The green Arabica beans contained higher oil content (15.16–16.85% dmb) than the Robusta beans (10.43–10.93% dmb). The obtained results are in accordance with the available literature, according to which the oil content in coffee beans ranges from ~5–15% [34,35,36] depending on the coffee variety, in favor of Arabica. In agreement with the fact that coffee oil is a relatively stable component during roasting [37], the decrease in oil content after roasting did not exceed 3% in all studied samples. Oliviera et al. [38] also reported a slight decrease in oil content in Arabica beans after roasting and a possible higher loss under more stringent processing conditions, while Budryn et al. [37], on the other hand, reported an increase in oil content after roasting of Robusta coffee beans due to the breakdown of carbohydrates, free amino acids and proteins and the evaporation of their degradation products. As Oliviera et al. [38] stated in their study, comparison of data in the literature should be done with caution because the oil content of coffee depends on many factors, such as variety, roasting parameters, number of defects in a lot, applied methodology for the determination of oil content, etc. [38].

**Figure 1:**
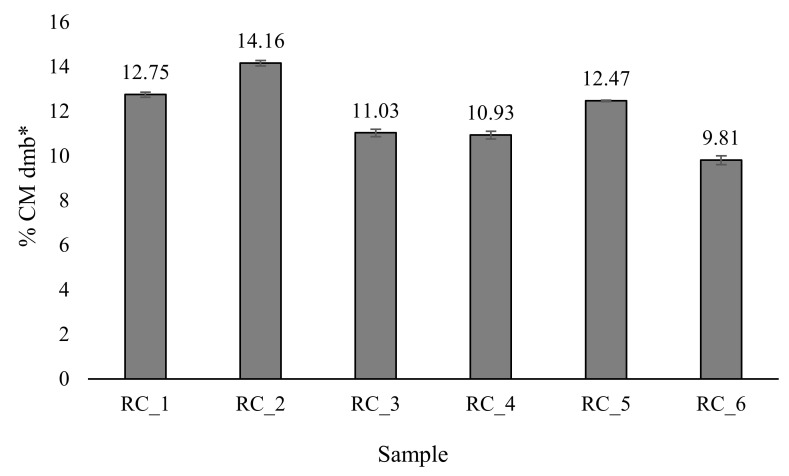
Melanoidin content in roasted (RC) coffee beans. CM—caramel melanoidins; * dmb—dry matter basis.

Green Arabica beans were also richer in sucrose than Robusta. The lowest sucrose content (2.50% dmb) was detected in Robusta sample GC_6, and the highest in Arabica sample GC_2 (5.85% dmb). The obtained results of sugar are similar to those reported in the available literature, according to which Knopp et al. [39] determined a sugar content of 7.79% dmb in green Arabica beans from Brazil, with sucrose as a major low molecular carbohydrate (7.07 dmb), while in the study of de Souza Gois Barbosa et al. [40], the sucrose content for green Arabica beans, also from Brazil, ranged from 5.13–7.76% with an average value of 6.66%. Apart from the variety and regional origin, sucrose content can be affected by maturity and pre- and post-harvest processing [41]. Sucrose contributes to the sweetness, color and aroma formation of the coffee brew and its higher content is associated with better cup quality [40,42]. Compared to green coffee beans, the sucrose content in roasted beans decreased in the range 84.40–98.27%. Although sucrose is a non-reducing sugar, it is hydrolyzed at high temperatures to glucose and fructose, which in turn are involved in Maillard reactions and caramelization [43]. Kocadaǧh et al. [44] reported the complete degradation of sucrose within 15 min after roasting Arabica beans at 220 °C, resulting in the accumulation of 5-hydroxymethylfurfural. Low water activity and high temperatures during roasting lead to the development of Maillard products, including melanoidins, between proteins and carbohydrates [45]. Melanoidins are a heterogenous group of brown-colored, water-soluble and nitrogenous polymeric macromolecular compounds whose complex structure is still unclear and strongly depends on food sources and processing conditions [46,47]. In the present study, the content of melanoidins in roasted beans was determined by a spectrophotometric method and the results are shown in Figure 1.

The highest melanoidin content was determined in sample RC_2 (14.16% CM dmb) and the lowest in sample RC_6 (9.81% CM dmb), which is in accordance with the sucrose content in green beans. According to Gniechwitz et al. [47], the melanoidins of Arabica coffee are in the molecular weight range of 3–22 kDa and are rich in non-carbohydrate and non-protein (~90%) chromophoric and phenolic moieties and contain less than 6% releasable amino acids and carbohydrates.

### 3.2. Bioactive Composition of Green and Roasted Coffee Beans

**Figure 2:**
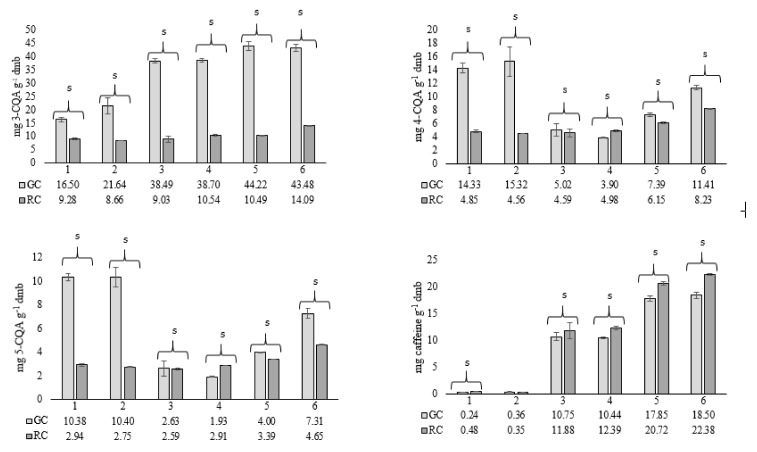
Content of individual phenolic compounds (3-CQA, 4-CQA and 5-CQA) and caffeine in green (GC) and roasted (RC) coffee beans. s—change in investigated parameter after roasting is significant (*p* < 0.05), determined by single-factor ANOVA.

The bioactive composition of green and roasted coffee beans was analyzed for their antioxidant capacity by monitoring their capacity to scavenge ABTS cation and DPPH free radicals, as well as by determination of total phenolic content (TPC), caffeine, 3-CQA, 4-CQA and 5-CQA content. Results are presented in Table 4 and Figure 2.

Green Robusta coffee beans exhibited higher antioxidant capacity and TPC than Arabica beans. The highest bioactive potential was observed in Robusta sample GC_6 which had an antioxidant capacity of 389.16 and 412.92 µmol TroloxE g^−1^ dmb and TPC of 68.67 mg GAE g^−1^ dmb, while the antioxidant capacity and TPC in green Arabica beans did not exceed 345 µmol TroloxE g^−1^ dmb and 50 mg GAE g^−1^ dmb, respectively. In the study by Song et al. [48], green Robusta beans were also richer in total phenolics (105.40 mg GAE g^−1^) compared to Arabica beans (65.92 mg GAE g^−1^). Similar findings were reported by Sacchetti et al. [49], where green Robusta beans had ~30% higher phenolic content than Arabica. The results of this study are also in agreement with the study of Tripetch and Borompichaichartkul [50], who determined a TPC of 40.14 mg GAE g^−1^ dmb in green Arabica beans. Further, the roasting process mainly resulted in an increase in antioxidant capacity, except for samples RC_1, RC_4 and RC_5, determined by the DPPH assay, and sample RC_6, determined by both DPPH and ABTS assays. The highest increase in antioxidant capacity after roasting was observed in samples RC_2 (ABTS assay, 27.58%) and RC_3 (DPPH, 10.57%). The roasting process had a determinable effect on TPC only in the sample RC_5, resulting in a decrease of 3.39%. There are conflicting results in the literature on the effects of the roasting process on TPC and the antioxidant capacity. Alkaltham et al. [34] reported a 13.59% and 16.66% decrease in TPC after microwave and oven roasting of Arabica beans, respectively, and a 34.76% and 37.63% loss in antioxidant capacity, respectively, because of phenolic degradation, autooxidation and polymerization processes during roasting. Del Castillo et al. [51] reported that the antioxidant capacity of coffee brews increased with the degree of roasting up to the medium-roasted sample but decreased in the dark-roasted sample. Sacchetti et al. [49] reported that the antioxidant capacity in brews of medium- and dark-roasted coffee was negatively affected by the intensity of the thermal process and appeared to be much more dependent on roasting severity than on the type of coffee. In the study by Bobková et al. [52], the antioxidant capacity of coffee increased after roasting to the light stage, and then decreased towards the dark-roast stage and, from a nutritional point of view, the most preferred coffees were those roasted at the light or medium stage, which guaranteed the highest content of antioxidants. The increase in antioxidant capacity after a certain point during the roasting process can be explained by the release of some phenolic compounds, such as hydroxycinnamic acid and quinic acid [34], as well as by the formation of Maillard products, such as melanoidins, that was discussed in the previous section. As has been proven, melanoidins possess certain antioxidant capacities due to low molecular weight compounds, such as chlorogenic acid and Maillard reaction products, linked to their core [53]. Gniechwitz et al. [47] suggested that the high antioxidant capacity of melanoidin fractions might be due to the high content of phenolic compounds, as indicated by the high UV absorbance of melanoidin fractions at 280 nm.

The content of 3-CQA, 4-CQA, 5-CQA and caffeine in green and roasted beans is presented in Figure 1. The highest content of 3-CQA and caffeine was found in green Robusta beans GC_5 (44.22 and 17.85 mg g^−1^ dmb, respectively) and GC_6 (43.98 and 18.50 mg g^−1^ dmb, respectively), while decaffeinated Arabica samples GC_1 and GC_2 had the highest content of 4-CQA (14.33 and 15.32 mg g^−1^ dmb, respectively) and 5-CQA (10.38 and 10.40 mg g^−1^ dmb, respectively). Roasting resulted in a decrease in caffeoylquinic acids in all samples, except for the sample RC_4 where the content of 4-CQA and 5-CQA increased. Although the decaffeinated samples had the highest content of 4-CQA and 5-CQA, roasting resulted in a ~70% decrease compared to the green beans and the final values were in the range of the other Arabica samples (4.56–4.98 mg g^−1^ dmb and 2.59–2.94 mg g^−1^ dmb, respectively), while the Robusta samples exhibited even higher values (6.15 and 8.23 mg g^−1^ dmb and 3.39 and 4.65 mg g^−1^ dmb, respectively). Dawidowicz and Typek [54] concluded that the roasting process of coffee beans leads to the degradation of chlorogenic acid isomers to volatile compounds and their transformation to quinic acid and its epimer and to four chlorogenic acid lactones, including basic and epimeric forms of 3- and 4-*O*-caffeoylquinic acid lactones. Further, roasting did not show a degradative effect on caffeine, as it is a thermostable compound [55], and its increase after roasting could be due to the loss of dry matter, as the results in the study are reported in dmb and thus were only corrected for loss of water, and/or due to the release of trapped carbon dioxide during roasting [56].

### 3.3. Bioactive Composition of Coffee Brews

The bioactive composition of prepared coffee brews (espresso, Turkish and filter) is presented in Table 5 and Table 6. 

The espresso brewing method resulted in the highest TPC, antioxidant capacity and content of caffeoylquinic acids and caffeine for all samples. In agreement with previous results on the bioactive composition of roasted beans, the Robusta sample RC_6 was characterized by the highest content of all bioactive parameters studied, regardless of the brewing method. The bioactive potential of coffee brews from decaffeinated samples RC_1 and RC_2 should be highlighted, proving that reduced-caffeine coffee brews with a rich bioactive composition and with pronounced antioxidant properties can be prepared. Among all investigated Arabica samples, the espresso brew of the decaffeinated sample RC_1 exhibited the highest antioxidant capacity (95.85 and 81.36 µmol TroloxE mL^−1^), TPC (16.48 mg GAE mL^−1^), 3-CQA (3.29 mg mL^−1^), 4-CQA (1.87 mg mL^−1^) and 5-CQA (1.13 mg mL^−1^) content and the lowest caffeine content (0.16 mg mL^−1^).

### 3.4. Sensory Analysis of Coffee Brews

**Figure 3:**
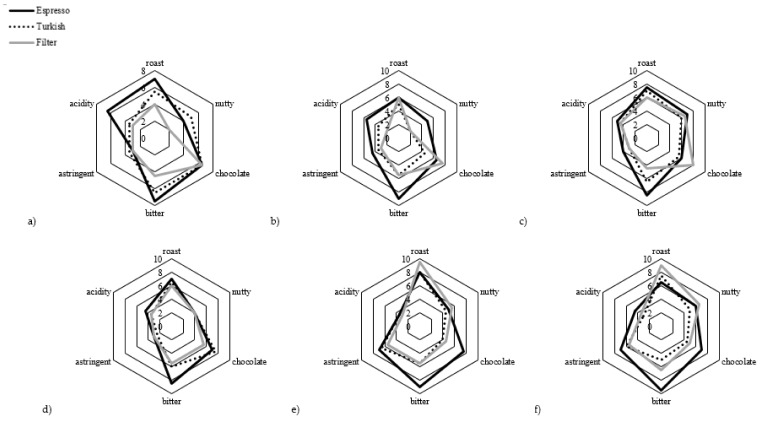
Results of the sensory evaluation of differently prepared coffee brews of (**a**) RC_1; (**b**) RC_2; (**c**) RC_3; (**d**) RC_4; (**e**) RC_5; (**f**) RC_6.

Sensory analysis of the coffee brews is shown in Figure 3 and Table 7. The statistical analysis of sensory evaluation is presented in the Supplementary Materials (Tables S1–S3). Bitterness, astringency and acidity were more pronounced in espresso than in Turkish and filter coffees, while the roasted aroma of Robusta samples mostly dominated in filter coffee. Robusta brews were found to be more astringent than Arabica regardless of the brewing method. The astringency is attributed to the chlorogenic acid content [57] and, therefore, Arabica had a milder taste due to lower content of these acids. The chocolate and nutty aromas were more dominant in the decaffeinated brews (RC_1 and RC_2) compared to regular coffees, but were also sensory scored as more bitter. According to Nebesny and Budryn [32], the nutty aroma of coffee is mainly due to pyrazines, which are formed intensively during the initial roasting stage, while the roasted aroma, besides pyrazines, is attributed to sulfides, thiofens, thiazols and furans. 

Descriptive attributes of the panelists are shown in Table 7. The widest range of aromas was observed in the Robusta sample RC_5, including tobacco, yellow fruits, dried fruits, herbal, woody and earthy, while ashy and citrus aromas were observed only in the sample RC_2. The woody and earthy aromas were perceived together only in the Robusta samples. According to Blank et al. [58], the typical earthy aroma impression of Robusta coffee is associated with the presence of 2-methylisoborneol. An interesting study by Bhumiratana et al. [59] showed that different sensory properties of coffee can trigger different emotions in coffee consumers. For example, coffee aroma, citrus and acidity elicited negative feelings, while cocoa aroma, tobacco, bitter, roast and burnt aromas generated positive emotions [59].

### 3.5. Formulation, Bioactive and Sensory Evaluation of Coffee Blends with Reduced Caffeine Content

Considering the increased consumer demand for coffee with reduced caffeine content due to negative health perceptions associated with caffeine and/or to avoid excessive caffeine intake, the main objective of the present study was to meet these requirements by formulation of coffee blends with reduced caffeine content, considering the results of the bioactive composition and sensory evaluation of the investigated Arabica and Robusta coffee beans. The Robusta sample RC_6 showed the highest bioactive potential in terms of TPC, antioxidant capacity (Table 4) and content of caffeoylquinic acids (Figure 2). Due to the highest caffeine content (Figure 2), it was used to formulate three coffee blends with reduced caffeine content and the final coffee blend formulations are presented in Table 8. 

The coffee blend with the highest caffeine content (CF_1; 20.44 mg g^−1^ dmb) consisted only of Robusta samples, RC_5 (30%) and RC_6 (70%), due to their great bioactive potential and satisfactory sensory properties. To obtain a lower caffeine content (15.44 mg g^−1^ dmb), 30% of RC_6 and 70% of RC_4 were blended, while the blend with the lowest caffeine content (5.55 mg g^−1^ dmb) was prepared with 50% of RC_6 and 50% of RC_1. The presented results regarding the bioactive composition indicate that coffee brews with reduced caffeine content may represent a significant source of phenolic compounds, mainly caffeoylquinic acids, with potent antioxidant properties.

**Figure 4:**
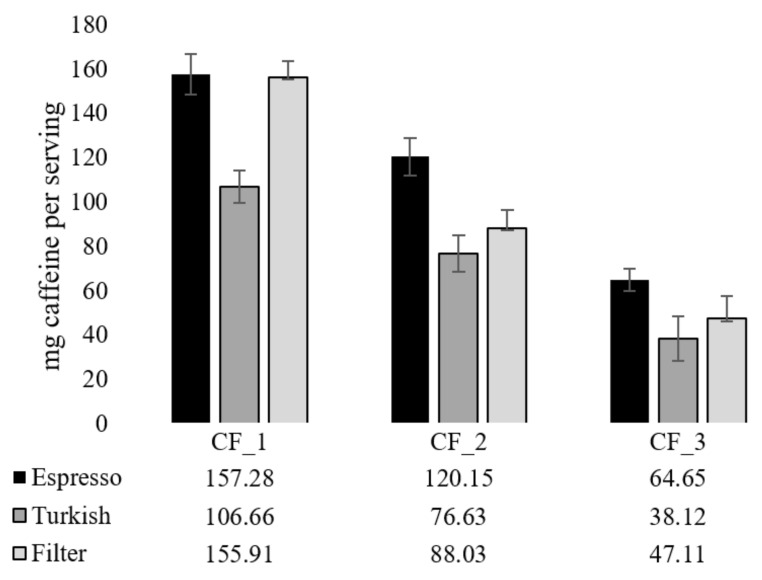
Caffeine intake per serving of formulated coffee blends (CF). Serving size for espresso is 30 mL, for Turkish coffee 60 mL, and for filter coffee 150 mL.

Caffeine intake per serving of each coffee blend is presented in Figure 4. 

The presented results demonstrate that two servings of CF_1 espresso and filter coffee can be consumed without concerns about exceeding the safe caffeine dose of 400 mg, a dose recognized by EFSA as safe for healthy adults [12], while other brews prepared from two other blends can be consumed in more servings. Further, if consuming three servings of espresso consisting of each new formulated coffee blend a day, daily caffeine intake would not exceed 400 mg (342.08 mg). The same was found for Turkish and filter coffee with caffeine content of 221.41 and 291.05 mg, respectively, for daily consumption of three cups of each coffee blend. The obtained results suggest that the formulation of coffee blends with reduced caffeine content was successful. 

**Figure 5:**
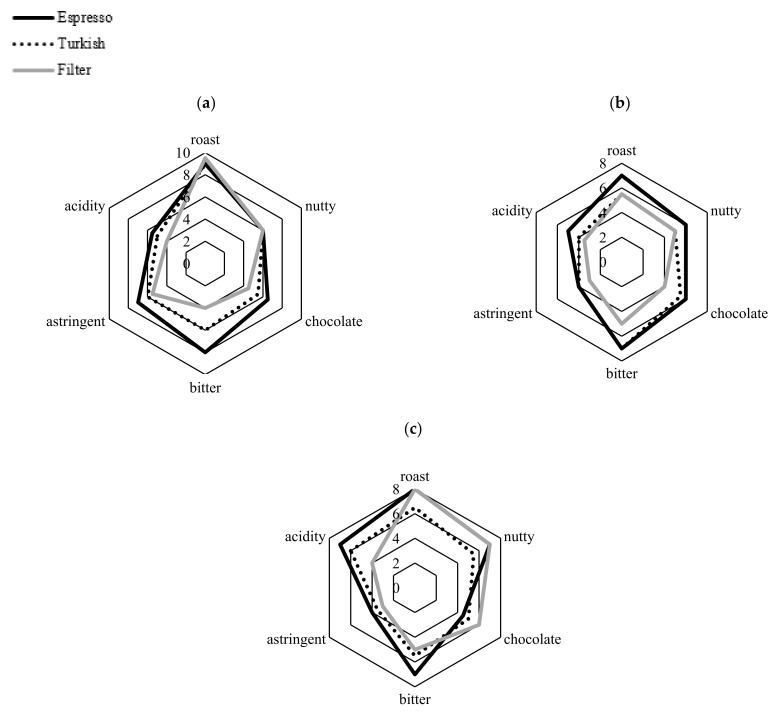
Results of the sensory evaluation of final coffee blends: (**a**) CF_1; (**b**) CF_2; (**c**) CF_3.

Sensory analysis of coffee blends is presented in Figure 5 and Table 9. The statistical analysis of sensory evaluation is presented in the Supplementary Materials (Tables S4–S6).

The sensory parameters of CF_1 are quite similar to those of its constituents, RC_5 and RC_6, and only a small decrease in bitterness was noted. The astringency of RC_6 was slightly reduced when blended with both RC_4 (CF_2) and RC_1 (CF_3) with a simultaneous increase in chocolate aroma. Herbal, earthy and woody aromas of RC_6 also came to the fore in blended forms (CF_2 and CF_3). The high acidity of RC_1 was maintained when blended with RC_6 (CF_3). According to the descriptive attributes of the panelists, the rich aromatic spectrum of the sample RC_5 was also perceived in CF_1. 

## Figures and Tables

**Table 1:** Species and region of investigated coffee beans.

Sample	Species	Country of Production	Coffee Region
GC_1	Arabica ^d1^	Mexico	Chiapas
GC_2	Arabica ^d2^	Colombia	Huila
GC_3	Arabica	Brazil	Minas Gerais
GC_4	Arabica	Costa Rica	Tarrazu Region
GC_5	Robusta	Guatemala	Volcanic San Marcos
GC_6	Robusta	Brazil	Minas Gerais

GC—green coffee beans; ^d1^ decaffeinated coffee beans, Swiss water^®^ process; ^d2^ decaffeinated coffee beans, sugar cane process.

**Table 2:** The parameters of roasting process for investigated coffee beans—initial roasting temperature (T_i_), final roasting temperature (T_f_) and time.

Sample	T_i_ (F)	T_f_ (F)	Time (min)
RC_1	355	410	10:50
RC_2	355	410	11:40
RC_3	375	410	14:15
RC_4	430	405	11:45
RC_5	360	420	10:30
RC_6	340	410	12:40

RC—roasted coffee beans.

**Table 3:** Dry matter, oil and sucrose content in green (GC) and roasted (RC) coffee beans.

Sample	Dry Matter (% of the Sample)		Oil Content (% dmb *)		Sucrose Content (% dmb)	
GC_1	91.09 ± 0.13	↑ 8.09% ^s^	15.16 ± 0.04	↓ 0.07%	4.05 ± 0.10	↓ 98.27% ^s^
RC_1	99.11 ± 0.01		15.15 ± 0.04		0.07 ± 0.02	
GC_2	90.67 ± 0.16	↑ 8.83% ^s^	15.73 ± 0.04	↑ 1.19% ^s^	5.85 ± 0.38	↓ 98.12% ^s^
RC_2	99.45 ± 0.23		15.92 ± 0.12		0.11 ± 0.01	
GC_3	91.68 ± 0.08	↑ 7.74% ^s^	16.85 ± 0.00	↓ 2.91% ^s^	5.05 ± 0.45	↓ 91.09% ^s^
RC_3	99.37 ± 0.39		16.36 ± 0.16		0.10 ± 0.00	
GC_4	89.54 ± 0.13	↑ 9.50% ^s^	15.35 ± 0.15	↓ 1.82% ^s^	5.19 ± 0.05	↓ 98.07% ^s^
RC_4	98.94 ± 0.03		15.07 ± 0.13		0.10 ± 0.01	
GC_5	90.49 ± 0.09	↑ 8.27% ^s^	10.93 ± 0.20	↓ 1.28% ^s^	3.96 ± 0.22	↓ 97.98% ^s^
RC_5	98.65 ± 0.31		10.79 ± 0.02		0.08 ± 0.02	
GC_6	90.43 ± 0.12	↑ 9.13% ^s^	10.43 ± 1.47	↓ 1.53% ^s^	2.50 ± 0.15	↓ 84.40% ^s^
RC_6	99.52 ± 0.05		10.27 ± 0.02		0.39 ± 0.01	

* dmb—dry matter basis; ↑—increase after roasting process in %; ↓—decrease after roasting process in %; ^s^—change in investigated parameter after roasting is significant (*p* < 0.05) determined by single-factor ANOVA.

**Table 4:** Antioxidant capacity and total phenolic content (TPC) of green (GC) and roasted (RC) coffee beans.

Sample	Antioxidant Capacity				TPC (mg GAE g^−1^ dmb)	
	ABTS (µmol TroloxE g^−1^ dmb *)		DPPH (µmol TroloxE g^−1^ dmb)			
GC_1	191.58 ± 17.92	↑ 26.92% ^s^	290.65 ± 6.11	↓ 7.04% ^s^	49.30 ± 0.93	↑ 2.67% ^s^
RC_1	262.16 ± 8.02		270.20 ± 1.60		50.65 ± 1.65	
GC_2	183.21 ± 15.96	↑ 27.58% ^s^	258.03 ± 13.89	↑ 8.88% ^s^	46.21 ± 1.04	↑ 14.57% ^s^
RC_2	252.97 ± 54.35		283.18 ± 1.16		54.09 ± 0.15	
GC_3	221.67 ± 22.36	↑ 5.05% ^s^	253.92 ± 8.33	↑ 10.57% ^s^	46.81 ± 0.22	↑ 6.53% ^s^
RC_3	233.45 ± 24.29		283.93 ± 2.83		50.08 ± 0.33	
GC_4	205.28 ± 6.56	↑ 13.39% ^s^	343.21 ± 26.00	↓ 18.83% ^s^	48.79 ± 0.02	↑ 7.33% ^s^
RC_4	237.02 ± 1.59		278.57 ± 10.70		52.65 ± 0.46	
GC_5	284.35 ± 13.33	↑ 22.16% ^s^	442.44 ± 54.21	↓ 16.13% ^s^	68.47 ± 2.69	↓ 3.39% ^s^
RC_5	365.32 ± 12.23		371.08 ± 16.16		66.15 ± 0.06	
GC_6	389.16 ± 13.91	↓ 3.60% ^s^	412.92 ± 14.57	↓ 5.65% ^s^	68.67 ± 1.73	↑ 5.63% ^s^
RC_6	375.52 ± 8.02		389.57 ± 20.73		72.77 ± 0.08	

* dmb—dry matter basis; TPC—total phenolic content; GAE—gallic acid equivalents; ↑—increase after roasting process in %; ↓—decrease after roasting process in %; ^s^—change in investigated parameter after roasting is significant (*p* < 0.05), determined by single-factor ANOVA.

**Table 5:** Antioxidant capacity and total phenolic content (TPC) of differently prepared coffee brews.

Sample		Antioxidant Capacity (µmol TroloxE mL^−1^)		TPC (mg GAE mL^−1^)
		DPPH	ABTS	
RC_1	Espresso	95.85 ± 0.33 ^ab^	81.36 ± 1.04 ^ab^	16.48 ± 0.10 ^ab^
	Turkish	26.72 ± 1.34 ^ac^	22.40 ± 0.30 ^ac^	3.87 ± 0.03 ^ac^
	Filter	11.94 ± 0.45 ^bc^	11.37 ± 0.59 ^bc^	1.88 ± 0.02 ^bc^
RC_2	Espresso	74.30 ± 0.17 ^ab^	66.36 ± 1.78 ^ab^	12.51 ± 0.09 ^ab^
	Turkish	30.82 ± 0.67 ^ac^	18.98 ± 0.15 ^ac^	3.95 ± 0.03 ^ac^
	Filter	12.09 ± 0.15 ^bc^	9.19 ± 0.59 ^bc^	1.82 ± 0.08 ^bc^
RC_3	Espresso	82.09 ± 0.83 ^ab^	79.13 ± 0.30 ^ab^	13.29 ± 0.17 ^ab^
	Turkish	29.33 ± 0.97 ^ac^	21.65 ± 0.45 ^ac^	3.92 ± 0.08 ^ac^
	Filter	15.82 ± 0.30 ^bc^	10.57 ± 0.20 ^bc^	1.93 ± 0.01 ^bc^
RC_4	Espresso	74.79 ± 1.16 ^ab^	66.15 ± 0.09 ^ab^	12.56 ± 0.18 ^ab^
	Turkish	27.69 ± 0.07 ^ac^	18.24 ± 0.89 ^ac^	3.61 ± 0.11 ^ac^
	Filter	14.63 ± 0.15 ^bc^	15.52 ± 0.20 ^bc^	2.08 ± 0.00 ^bc^
RC_5	Espresso	82.59 ± 1.16 ^ab^	65.41 ± 0.24 ^ab^	13.31 ± 0.13 ^ab^
	Turkish	34.25 ± 0.97 ^ac^	27.74 ± 0.30 ^ac^	5.05 ± 0.09 ^ac^
	Filter	16.79 ± 0.22 ^bc^	10.18 ± 0.59 ^bc^	2.44 ± 0.02 ^bc^
RC_6	Espresso	129.60 ± 1.24 ^ab^	111.59 ± 0.21 ^ab^	19.86 ± 0.34 ^ab^
	Turkish	31.34 ± 1.04 ^ac^	25.81 ± 0.45 ^ac^	4.96 ± 0.08 ^ac^
	Filter	11.94 ± 0.45 ^bc^	15.13 ± 0.40 ^bc^	2.96 ± 0.10 ^bc^

TPC—total phenolic content; GAE—gallic acid equivalents; means denoted with the same superscript letter within the same sample (RC_1–RC_6) including 3 techniques of preparation (espresso, Turkish, filter) are significantly different (*p* < 0.05), determined by one-way ANOVA and post hoc analysis (Tukey’s HSD test).

**Table 6:** Content of 3-CQA, 4-CQA, 5-CQA and caffeine in differently prepared coffee brews.

Sample		3-CQA	4-CQA	5-CQA	Caffeine
		mg mL^−1^			
RC_1	Espresso	3.29 ± 0.01 ^ab^	1.87 ± 0.03 ^ab^	1.13 ± 0.04 ^ab^	0.16 ± 0.03 ^ab^
	Turkish	0.72 ± 0.00 ^ac^	0.40 ± 0.01 ^ac^	0.25 ± 0.02 ^ac^	0.08 ± 0.00 ^ac^
	Filter	0.40 ± 0.02 ^bc^	0.22 ± 0.01 ^bc^	0.13 ± 0.02 ^bc^	0.03 ± 0.01 ^bc^
RC_2	Espresso	2.39 ± 0.07 ^ab^	1.32 ± 0.09 ^ab^	0.81 ± 0.08 ^ab^	0.29 ± 0.03 ^ab^
	Turkish	0.78 ± 0.03 ^ac^	0.42 ± 0.02 ^ac^	0.27 ± 0.02 ^ac^	0.05 ± 0.00 ^a^
	Filter	0.35 ± 0.02 ^bc^	0.19 ± 0.05 ^bc^	0.12 ± 0.01 ^bc^	0.05 ± 0.00 ^b^
RC_3	Espresso	2.53 ± 0.06 ^ab^	1.34 ± 0.05 ^ab^	0.81 ± 0.09 ^ab^	3.91 ± 0.10 ^ab^
	Turkish	0.72 ± 0.04 ^ac^	0.38 ± 0.02 ^ac^	0.24 ± 0.04 ^ac^	1.10 ± 0.02 ^ac^
	Filter	0.39 ± 0.01 ^bc^	0.20 ± 0.00 ^bc^	0.13 ± 0.01 ^bc^	0.64 ± 0.03 ^bc^
RC_4	Espresso	2.90 ± 0.04 ^ab^	1.47 ± 0.03 ^ab^	0.89 ± 0.05 ^ab^	3.63 ± 0.08 ^ab^
	Turkish	0.78 ± 0.03 ^ac^	0.38 ± 0.07 ^ac^	0.24 ± 0.01 ^ac^	0.90 ± 0.07 ^ac^
	Filter	0.45 ± 0.03 ^bc^	0.22 ± 0.02 ^bc^	0.14 ± 0.01 ^bc^	0.53 ± 0.03 ^bc^
RC_5	Espresso	2.37 ± 0.06 ^ab^	1.48 ± 0.04 ^ab^	0.87 ± 0.02 ^ab^	4.94 ± 0.05 ^ab^
	Turkish	0.87 ± 0.02 ^ac^	0.54 ± 0.01 ^ac^	0.31 ± 0.01 ^ac^	1.83 ± 0.07 ^ac^
	Filter	0.41 ± 0.02 ^bc^	0.24 ± 0.00 ^bc^	0.14 ± 0.01 ^bc^	0.86 ± 0.04 ^bc^
RC_6	Espresso	3.10 ± 0.05 ^ab^	2.09 ± 0.02 ^ab^	1.14 ± 0.01 ^ab^	8.65 ± 0.11 ^ab^
	Turkish	0.61 ± 0.04 ^ac^	0.42 ± 0.01 ^ac^	0.24 ± 0.03 ^ac^	1.71 ± 0.04 ^ac^
	Filter	0.40 ± 0.01 ^bc^	0.26 ± 0.01 ^bc^	0.15 ± 0.01 ^bc^	1.17 ± 0.03 ^bc^

Means denoted with the same superscript letter within the same sample (RC_1–RC_6) including 3 techniques of preparation (espresso, Turkish, filter) are significantly different (*p* < 0.05), determined by one-way ANOVA and post hoc analysis (Tukey’s HSD test).

**Table 7:** Descriptive attributes of coffee brews.

Sample	Descriptive Attributes
RC_1	tobacco, dried fruits, earthy
RC_2	ashy, cinnamon, yellow fruits, citrus
RC_3	yellow fruits, dried fruits, herbal, woody
RC_4	tobacco, yellow fruits, dried fruits, herbal, woody
RC_5	tobacco, yellow fruits, dried fruits, herbal, woody, earthy
RC_6	dried fruits, herbal, woody, earthy

**Table 8:** Bioactive composition of coffee blend (CB) formulations.

Sample	Formulation	Antioxidant Capacity (µmol TroloxE g^−1^ dmb *)		TPC (mg GAE g^−1^ dmb)	3-CQA	4-CQA	5-CQA	Caffeine
		ABTS	DPPH		(mg g^−1^ dmb)			
CF_1	70% RC_6 30% RC_5	352.88 ± 10.79 ^ab^	406.27 ± 6.84 ^ab^	68.60 ± 1.04 ^ab^	11.87 ± 0.44 ^ab^	6.95 ± 0.24 ^ab^	3.55 ± 0.26 ^a^	20.44 ± 0.68 ^ab^
CF_2	70% RC_4 30% RC_6	300.36 ± 8.09 ^ac^	317.18 ± 12.32 ^ac^	58.27 ± 0.61 ^ac^	10.69 ± 0.36 ^ac^	5.49 ± 0.20 ^ac^	3.22 ± 0.19 ^b^	15.44 ± 0.7 ^ac^
CF_3	50% RC_6 50% RC_1	242.10 ± 7.92 ^bc^	276.08 ± 12.41 ^bc^	51.70 ± 0.71 ^bc^	8.82 ± 1.08 ^bc^	4.54 ± 0.61 ^bc^	2.25 ± 0.23 ^ab^	5.55 ± 0.7 ^bc^

* dmb—dry matter basis; TPC—total phenolic content; GAE—gallic acid equivalents; determined in the ethanolic extracts prepared as described in Section 2.2.4; means denoted with the same superscript letter are significantly different (*p* < 0.05), determined by one-way ANOVA and post hoc analysis (Tukey’s HSD test).

**Table 9:** Descriptive attributes of coffee blends.

Sample	Descriptive Attributes
CF_1	woody, earthy, dried fruits, yellow fruits, herbal, tobacco
CF_2	yellow fruits, herbal, woody, earthy
CF_3	dried fruits, herbal, woody, earthy, tobacco

## Data Availability

Not applicable.

## Supplementary Materials

The following are available online, Table S1: The results of the statistical analysis of the sensory evaluation of espresso coffee brews made from roasted coffee beans, Table S2: The results of the statistical analysis of the sensory evaluation of Turkish coffee brews made from roasted coffee beans, Table S3: The results of the statistical analysis of the sensory evaluation of filter coffee brews made from roasted coffee beans, Table S4: The results of the statistical analysis of the sensory evaluation of espresso brews made from final coffee blends, Table S5: The results of the statistical analysis of the sensory evaluation of Turkish brews made from final coffee blends, Table S6: The results of the statistical analysis of the sensory evaluation of filter brews made from final coffee blends.
