# Supplementary material for: Antioxidant and Sensory Assessment of Innovative Coffee Blends of Reduced Caffeine Content
Source: Molecules. 2022 Jan 10;27(2):448. doi: 10.3390/molecules27020448 (PMC8778917; doi:10.3390/molecules27020448)
Supplement: Supplementary file 1 [file molecules-27-00448-s001.zip › molecules-1525898-supplementary.pdf]

Supplementary Materials

Antioxidant and Sensory Assessment of Innovative Coffee Blends of Reduced Caffeine Content

Danijela Šeremet [1], Patricia Fabečić [1], Aleksandra Vojvodić Cebin [1], Ana Mandura Jarić [1], Robert Pudić [2] and Draženka Komes [1,*]

1 Faculty of Food Technology and Biotechnology, University in Zagreb, Pierottijeva Street 6, 10000 Zagreb, Croatia; dseremet@pbf.hr (D.Š.); pfabecic@pbf.hr (P.F.); avojvodic@pbf.hr (A.V.C.); amandura@pbf.hr (A.M.J.)

2 Quahwa Coffee Roastery & Coffee Shop, Nikola Tesla Street 9/1, 10000 Zagreb, Croatia; robert.pudic@quahwa.hr

* Correspondence: dkomes@pbf.hr

Table S1. The results of the statistical analysis of the sensory evaluation of espresso coffee brews made from roasted coffee beans.

Sample	Roast	Nutty	Chocolate	Bitter	Astringent	Acidity
RC_1	7	4^{ab}	6.5	7.5^{abc}	3^{abc}	6.5^{abcd}
RC_2	6^{ab}	5^{c}	6.5	9^{a}	4.5^{adef}	5.5^{e}
RC_3	7.5^{a}	7^{acde}	6^{a}	8.5	4^{gh}	5^{af}
RC_4	7	4^{df}	7	8.5	3^{dij}	4.5^{b}
RC_5	8^{bc}	5^{e}	7.5^{a}	9^{b}	7^{begi}	3.5^{cef}
RC_6	6.5^{c}	6^{bf}	7	9.5^{c}	7^{cfhj}	4.5^{d}

Means denoted with the same superscript letter within the same column are significantly different ($p<0.05$) determined by one-way ANOVA and post-hoc analysis (Tukey's HSD test).

Table S2. The results of the statistical analysis of the sensory evaluation of Turkish coffee brews made from roasted coffee beans.

Sample	Roast	Nutty	Chocolate	Bitter	Astringent	Acidity
RC_1	5.5^{abc}	5^{a}	6.5^{abc}	6.5^{a}	3.5^{ab}	3.5
RC_2	5^{defg}	2^{abcde}	4.5^{ad}	5.5	3.5^{cd}	3.5
RC_3	7^{ad}	6^{bfg}	5.5^{ef}	6.5^{b}	3^{ef}	4.5^{a}
RC_4	6.5^{eh}	4^{cf}	7.5^{degh}	6	2.5^{gh}	3.5
RC_5	8^{bfh}	4.5^{dg}	4^{bfg}	5.5	6.5^{aceg}	3.5
RC_6	7.5^{cg}	5^{e}	4.5^{ch}	5^{ab}	6^{bdfh}	3^{a}

Means denoted with the same superscript letter within the same column are significantly different ($p<0.05$) determined by one-way ANOVA and post-hoc analysis (Tukey's HSD test).

Table S3. The results of the statistical analysis of the sensory evaluation of filter coffee brews made from roasted coffee beans.

Sample	Roast	Nutty	Chocolate	Bitter	Astringent	Acidity
RC_1	4^{abcde}	2^{abcd}	6.5^{abc}	4.5^{a}	3^{ab}	3^{a}
RC_2	6^{afg}	2^{efgh}	7.5^{def}	5.5	3^{cd}	2^{bcd}
RC_3	6^{bhi}	6.5^{aei}	8^{aghi}	4.5^{b}	3^{ef}	4.5^{abe}
RC_4	6^{cjk}	4^{bfijk}	5.5^{dg}	5.5	3^{gh}	3.5^{c}
RC_5	9.5^{dfhj}	5.5^{cgj}	4.5^{beh}	5.5	5.5^{aceg}	3^{e}
RC_6	9^{egik}	6.5^{dhk}	5^{cfi}	6.5^{ab}	5.5^{bdfh}	3.5^{d}

Means denoted with the same superscript letter within the same column are significantly different ($p<0.05$) determined by one-way ANOVA and post-hoc analysis (Tukey's HSD test).

Table S4. The results of the statistical analysis of the sensory evaluation of espresso brews made from final coffee blends.

Sample	Roast	Nutty	Chocolate	Bitter	Astringent	Acidity
CF_1	9^{a}	6	6.5^{a}	8	7^{ab}	5.5^{a}
CF_2	7^{a}	6	6^{b}	7	4^{a}	5^{b}
CF_3	8	7	4.5^{ab}	7	4^{b}	7^{ab}

Means denoted with the same superscript letter within the same column are significantly different ($p<0.05$) determined by one-way ANOVA and post-hoc analysis (Tukey's HSD test).

Table S5. The results of the statistical analysis of the sensory evaluation of Turkish brews made from final coffee blends.

Sample	Roast	Nutty	Chocolate	Bitter	Astringent	Acidity
CF_1	9^{ab}	6	5.5	6	6^{ab}	5
CF_2	5.5^{a}	5	5.5	7^{a}	4^{a}	4^{a}
CF_3	6.5^{b}	5.5	5	5.5^{a}	3.5^{b}	6^{a}

Means denoted with the same superscript letter within the same column are significantly different ($p<0.05$) determined by one-way ANOVA and post-hoc analysis (Tukey's HSD test).

Table S6. The results of the statistical analysis of the sensory evaluation of filter brews made from final coffee blends.

Sample	Roast	Nutty	Chocolate	Bitter	Astringent	Acidity
CF_1	9.5^{a}	6	4.5^{a}	4	5.5^{ab}	4
CF_2	5.5^{ab}	5^{a}	4^{b}	5	3^{a}	3.5
CF_3	8^{b}	7^{a}	6^{ab}	5	3^{b}	4

Means denoted with the same superscript letter within the same column are significantly different ($p<0.05$) determined by one-way ANOVA and post-hoc analysis (Tukey's HSD test).
